# Characterization of multispecies microbial communities at beef and pork processing plants and their impact on pathogen stress tolerance

**DOI:** 10.3389/fmicb.2025.1605719

**Published:** 2025-07-02

**Authors:** Rong Wang, Sapna Chitlapilly Dass, Vignesh Palanisamy, You Zhou, Tatum Katz, Joseph M. Bosilevac

**Affiliations:** ^1^US Department of Agriculture, Agricultural Research Service, U.S. Meat Animal Research Center, Clay Center, NE, United States; ^2^Department of Animal Science, Texas A&M University, College Station, TX, United States; ^3^Center for Biotechnology, University of Nebraska–Lincoln, Lincoln, NE, United States

**Keywords:** sanitization, biofilm, beef and pork processing, *Salmonella enterica*, *E. coli* O157: H7, metagenomics

## Abstract

**Introduction:**

*E. coli* O157: H7 and *Salmonella enterica* are major foodborne pathogens. Biofilm formation may potentially contribute to product contamination by these pathogens at meat processing facilities. Further, pathogen stress tolerance may vary significantly due to the interactions with the multispecies microbial community at meat plants, which may be affected by processing activity, animal species, and the local selective pressure caused by sanitization practices.

**Methods:**

We characterized natural microorganisms collected from floor drains at various areas at three beef plants and two pork plants and analyzed their impact on pathogen sanitizer tolerance.

**Results:**

The pathogen strains were able to integrate efficiently into the multispecies community attached on contact surfaces even under low temperatures (7°C and 15°C) commonly seen in processing facilities. Cell density of the adhered *S. enterica* (4.9 to 6.3 log_10_ CFU/chip) was higher than *E. coli* O157: H7 (3.2–5.2 log_10_ CFU/chip). Contact surface materials and meat plant types did not affect surface attachment of either pathogen species. A multi-component sanitizer exhibited high efficiency that reduced the adhered pathogen cells in most samples to a non-enumerable level. However, overall higher survival and post-sanitization recovery of pathogen cells were observed in the treated pork plant samples than those in the beef plant samples. Scanning electron microscope analysis showed that the contact surface topography may impact the morphology of the attached microcolonies and bacterial tolerance. Metagenomic analysis of the multispecies bacterial communities showed that *Pseudomonadaceae, Halomonadaceae* and *Enterobacteriaceae* were the three most abundant families across all samples. No significant difference in genus compositions between the beef and pork plants or among the drain areas was observed. However, variations in the percentages of species’ relative abundance were observed among the samples.

**Discussion:**

The multispecies microbial community at the processing plants and the resulting interspecies interactions could influence the tolerance level of the pathogens integrated into the community. Therefore, research reports on sanitization processes and the resulting pathogen inactivation and prevalence prevention that are described for the different types of processing facilities should be analyzed on a case-to-case basis.

## Introduction

In the meat industry, product contamination by foodborne pathogens such as *Escherichia coli* O157: H7 and *Salmonella enterica* is a serious public health concern and may cause significant financial and societal losses. Even though animal hides have been deemed as the main contamination source at beef and pork processing plants, available results suggest that bacterial biofilm formation and subsequent enhanced pathogen stress tolerance may play an important role in meat contamination events (Wang et al., 2014, 2017; Visvalingam et al., 2016; Yang et al., 2015).

It has been well reported that meat processing facilities harbor a wide variety of microorganisms that persist and form multispecies biofilms in the plants (Xu et al., 2025). Such multispecies communities in turn may provide an ecological niche for the pathogens to better colonize and gain tolerance against sanitization. Since floor drains collect all materials flowing through the processing plants, they contain the microorganisms present throughout the nearby processing area. Our previous study of multispecies biofilms at a beef processor experiencing an increased *E. coli* O157: H7 prevalence showed these mixed biofilms protected *E. coli* O157: H7 to a significantly greater extent than the biofilms recovered from a control plant not experiencing similar contamination problems (Chitlapilly Dass et al., 2020).

Due to the multi-layered, 3-dimensional (3-D) biofilm structure and the well-expressed bacterial polymeric extracellular substances (EPS), available studies have shown that complete removal and elimination of mature *E. coli* O157: H7 and *S. enterica* biofilms in the meat processing plants are difficult to achieve using common sanitizers (Corcoran et al., 2014; Van der Veen and Abee, 2011). One novel approach to address this challenge is the multicomponent sanitizer design that takes advantage of the synergistic effects by combining multiple chemical reagents with different functional mechanisms and has shown enhanced effectiveness on biofilm control (DeQueiroz and Day, 2007; Wang et al., 2020, 2021). However, most studies have focused on single-species pathogen biofilms and did not take into consideration that pathogens can be harbored in natural mixed biofilms which are commonly seen in commercial facilities. As we demonstrated previously (Chitlapilly Dass et al., 2020), sanitizer effectiveness can be affected by the interactions between the pathogens and the natural multispecies microorganisms that vary depending on the processing plant types, activities, and locations. Furthermore, in addition to biofilm cell inactivation, post-sanitization pathogen survival control and recolonization prevention are all essential for reducing biofilm–related product contamination.

Therefore, in the present study, we characterized and compared the multispecies microorganisms in floor drain samples collected from three beef plants and two pork plants. Amplicon sequencing was performed to analyze the composition of the microbial communities at the two different types of processing facilities. Further, surface attachment and biofilm formation by the drain samples and pathogen (*E. coli* O157: H7 and *S. enterica*) adherence within the mixture was investigated in an attempt to understand how the various microbial communities might affect pathogen survival and prevalence after the treatment by a multicomponent sanitizer used in the meat processing industry.

## Materials and methods

### Floor drain sample collection and characterization

Three beef processing plants (designated Plant A, B, and C) and two pork processing plants (designated Plant D and E) were visited to collect bacterial samples from floor drains. Floor drains located at areas of the processing floor, cooler, and hotbox of the beef plants, and fabrication room and cooler of the pork plants were sampled using cellulose sponges (Speci-sponge; Nasco, Atkinson WI) to collect bacteria and biofilms as previously described (Wang et al., 2022). Briefly, the drain covering grate was removed then an area of ∼500 cm^2^ was vigorously swabbed with the sponge, turning it over halfway through the process. The underside of the grate and interior surfaces were sampled to collect the attached microorganisms. Sponges were sealed in the whirl-pak bags and transported to the laboratory on wet ice in a cooler. To ensure an adequate sample was obtained from each drain, each sample was thoroughly hand massaged then portions were removed and serially diluted to measure the levels of aerobic plate count (APC), total mesophile count (TMC), and psychrophilic bacteria (PB), as well as *Enterobacteriaceae* (EB), coliforms (CF) and *E. coli* (EC) using appropriate Petrifilm (3 M, St Paul, MN), incubated 24 h at 37°C for APC, EB, CF and EC; 48 h at 30°C for TMC and 10 days at 7°C for PB as described previously (Chitlapilly Dass et al., 2020).

### Culture conditions for drain samples and the *Salmonella enterica* and *Escherichia coli* O157: H7 strains

To best maintain the original microbial composition of the floor drain samples and expand the sample volume for experimental repetition, all selected samples confirmed to be free of *E. coli* O157: H7 and *S. enterica* were enriched in Luria Broth containing no salt (LB-NS) medium at 7°C (to simulate typical processing plant environmental temperature) for 5 days except for the pork plant fabrication room samples which were enriched at 15°C (the observed mean temperature at time of collection). After enrichment, equal volumes of each drain sample collected from similar areas of each plant were combined as a pool to represent the multispecies microorganisms commonly present in the area (Table 1).

**Table 1:** Survival and post-sanitization prevalence of *S. enterica* and *E. coli* O157: H7 cells in mixed biofilms on stainless steel or tile surface after the foam or fog treatment using the multicomponent Deep-Clean sanitizer.

Sample	Samples	Foam				Fog	
Name	Pooled	SS		Tile		SS	
		*S. enterica*	O157	*S. enterica*	O157	*S. enterica*	O157
A1-processing floor	5a, 6a	ND (−)	ND (−)	ND (−)	ND (−)	ND (−)	ND (−)
A2-cooler	7a, 8a, 9a	ND (−)	ND (−)	ND (−)	ND (−)	1.6 ± 1.4	ND (−)
A3-hotbox	10a, 11a	ND (−)	ND (−)	ND (−)	ND (−)	ND (−)	ND (−)
B1-processing floor	1b, 6b	ND (−)	ND (−)	ND (−)	ND (−)	ND (−)	ND (−)
B2-cooler	7b, 8b, 9b	ND (−)	ND (−)	ND (−)	ND (−)	ND (−)	ND (−)
B3-hotbox	10b, 11b	ND (−)	ND (−)	ND (−)	ND (−)	1.7 ± 1.5	ND (−)
C1-processing floor	1c, 2c, 11c	ND (−)	ND (−)	ND (−)	ND (−)	ND (−)	ND (−)
C2-cooler	12c, 13c, 14c	ND (−)	ND (−)	ND (−)	ND (−)	ND (−)	ND (−)
C3-hotbox	15c, 17c, 18c	ND (−)	ND (−)	ND (−)	ND (−)	0.9 ± 1.6	ND (−)
D1-cooler	1d, 2d, 4d, 5d, 6d	ND (−)	ND (+)	ND (−)	ND (+)	0.7 ± 1.3	ND (−)
D2-fabrication	9d, 12d, 13d, 14d	ND (−)	ND (+)	ND (−)	ND (−)	2.5 ± 0.3	ND (−)
E1-cooler	1e, 2e, 3e, 4e	ND (−)	ND (+)	ND (−)	ND (−)	2.9 ± 0.5	ND (−)
E2-fabrication	7e, 8e, 9e, 11e, 12e	ND (−)	ND (+)	ND (+)	ND (+)	1.6 ± 1.4	ND (−)

Data of viable bacteria after sanitization are shown as log_10_ CFU/chip ± standard deviation (*n* = 3). ND: not detected, indicating that the level of viable cells was lower than the limit of detection; (+): positive bacterial recovery growth in enriched nonenumerable (ND) samples. (−): negative bacterial recovery growth in enriched nonenumerable (ND) samples.

To investigate pathogen colonization and stress tolerance in floor drain mixed biofilms, an *E. coli* O157: H7 cocktail and a *S. enterica* cocktail were prepared by mixing equal volumes of overnight cultures of five *E. coli* O157: H7 strains (MARC-110, 141, 144, 168, 170) and five *S. enterica* strains of various serovars (Anatum, Dublin, Montevideo, Newport, Typhimurium), respectively. All pathogen strains were previously isolated from beef trim samples at commercial meat plants (Wang et al., 2014, 2017).

### Biofilm formation and sanitizer treatment

The pooled representative drain samples were used for biofilm development and sanitization studies conducted on stainless steel (SS) or tile surfaces following established protocols (Chitlapilly Dass et al., 2020). Briefly, biofilms were developed by incubating the SS (18 × 18 × 2 mm, 2B brushed finish) or tile (40 × 23 × 6 mm, porcelain mosaic) chips in the enriched and pooled drain samples with the co-inoculation of the *E. coli* O157: H7 or *S. enterica* cocktail that was inoculated into the pooled drain samples at a 1:100 ratio for 5 days at 15°C (pork plant fabrication room samples) or 7°C (all other samples). Biofilm formation by the *E. coli* O157: H7 or *S. enterica* cocktail only at 7°C or 15°C were included as control samples.

The microbial communities harboring the *E. coli* O157: H7 or *S. enterica* cocktail attached on SS or tile surfaces were treated with a multi-component sanitizer Deep-Clean (PSSI Chemical Innovations, Kieler, WI), a mixture of surfactants, solvents, alkaline builders and oxygen bleach. The active ingredients include hydrogen peroxide, alkyl dimethyl benzyl ammonium chloride (C12-16), isobutyl alcohol, diethylene glycol monobutyl ether, ethanol, and potassium hydroxide. The sanitizer working solution was prepared following the manufacturer’s instructions and applied to the biofilm samples harboring the pathogen cells with either a foam or fog treatment. For the foam treatment, the sanitizer foam was generated using an air foam generator (Innovative Cleaning Equipment, Inc., Grand Rapids, MI), and sufficient amounts of foam were applied to ensure that the entire chip surface was fully covered with the sanitizer for 1 h even after the foam turned into the liquid state after the prolonged exposure period. The 3-h fog treatment was conducted using 1 L of the prepared sanitizer working solution and applied with a fog generator (Curtis Dyna-Fog Ltd., Westfield, IN) to fill a sealed 650 cubic feet space (18.4 m^3^) containing the chips colonized with the mixed biofilms harboring the pathogens. Samples treated with sterile water rinse only were included as positive controls.

At the end of each treatment, the sanitizer activity was neutralized with sterile Dey/Engley broth (Becton Dickinson, Franklin Lakes, NJ) supplemented with 0.3% soytone and 0.25% sodium chloride. Biofilm cells on each chip were harvested by 1 min sonication followed by vigorous surface scraping, 5 times each surface, using a cell scraper (Corning Inc., Corning, NY). Cells on the scraper were further collected by dipping and stirring in the Dey/Engley broth in the sample tube, and each side of the chip surface was also further rinsed 3 times using a pipette tip with 1 mL Dey/Engley broth in the tube each time. The harvested biofilm cells in the broth were vigorously vortexed for 2 min, then further diluted in sterile Dey/Engley broth for plating. Total bacteria, *E. coli* O157: H7 and *S. enterica* cells in mixed biofilms of the positive controls or their survival in the sanitizer-treated samples were measured by plating the samples onto tryptone soya agar plates (TSA, Becton Dickinson, Franklin Lakes, NJ), ChromAgar O157 agar plates (DRG International, Springfield, NJ), and xylose lysine deoxycholate agar plates (XLD, Oxoid Ltd., Hampshire, England), respectively, as previously described (Wang et al., 2021).

The Dey/Engley broth containing the harvested sanitizer-treated biofilm cells was further incubated overnight at 37°C and then streaked onto ChromAgar O157 or XLD agar plates to confirm pathogen recovery growth and prevalence after sanitization if no enumerable bacterial cells were observed after the treatment as previously described (Wang et al., 2021).

### Scanning electron microscopy

Selected biofilm samples on chips treated with sterile water (positive control) were fixed with 2.5% glutaraldehyde in 0.1 M cacodylate buffer for 2 h at room temperature. Samples were rinsed briefly and postfixed with 1% osmium tetroxide for 1 h then processed for dehydration through an ethanol series (30 to 100%) and air dried. The chips were then mounted onto the SEM stubs, placed in a 42°C vacuum oven overnight, and sputter coated with a thin layer (8- to 10-nm thick) of chromium with sputter coater (Desk V, Denton Vacuum, Moorestown, NJ). The coated samples were examined under a field-emission scanning electron microscope (S-4700, Hitachi, Tokyo, Japan) to directly observe the chip surface texture for its potential impact on the morphology and tolerance of biofilms formed by the multispecies microorganisms.

### DNA extraction and 16S rRNA gene amplicon-based sequencing

DNA extraction and purification were performed using the enriched and pooled drain samples for amplicon sequencing based on the variable region V4 of the 16Sr RNA gene as previously described. Briefly, primers used were 15F (5’-GTGCCAGCMGCCGCGGTAA-3′) and 806R (5’-GGACTACHVGGGTWTCTAAT3’), flanking the 515 and 806 regions. Barcodes were attached to the 806R primers. Library preparation and 2 × 250 bp paired-end sequencing was carried out using the Illumina® MiSeq® platform by Novogene Co. Ltd. (Sacramento, CA) as described previously (Chitlapilly Dass et al., 2020).

### Statistical and bioinformatics analysis

Experiments of biofilm formation or sanitizer treatment were conducted at least twice with replicated samples using fresh cultures each time. Bacterial density was measured and expressed as logarithmic cell counts (log_10_ CFU/chip). Statistical analysis was performed in the R statistical software version 4.4.1 “Race for Your Life” (R Core Team, 2024). To compare the attachment of *S. enterica* and *E. coli* O157: H7 cells across beef and pork plants and contact surface types, a three-way ANOVA with all interactions was run followed by a Tukey–Kramer post-hoc comparison with a family-wise alpha of 0.05 using package multcompView (Graves et al., 2024).

To analyze the 16S rRNA sequencing results, FastQC was used to assess the quality of raw fastq datasets.^1^ The qiime2 pipeline^2^ was used to process all of the samples. The raw fastq sequences of all samples were imported into qiime2 for taxon analysis. DADA2 was used for removing the low-quality sequences based on the quality with 210 set as the truncation length to obtain the number of sequences for each OTUs and the representative sequences. Taxonomy assignment was performed for the representative sequences using the SILVA 138 release database’s pre-trained classifiers for the 99 percent OTUs.^3^ For further statistical analysis, BIOM file was exported and was converted into a tab-delimited format using the biom convert command in qiime2 to generate the OTU table. Downstream statistical analyses were carried out using R.

## Results

### Levels of indicator bacteria groups in floor drain samples

To assess the quality and composition of the drain samples, general indicator organism groups in each original (pre-enrichment) sample were measured. Aerobic plate count (APC), total mesophiles count (TMC), psychrophiles (PSY), *Enterobacteriaceae* (EB), coliforms (CF), and *E. coli* (EC) were all enumerated. All values were log-transformed and presented as log_10_ CFU/100cm^2^ in Table 2 and the Supplementary Table 2.

**Table 2:** Measurement (log_10_ CFU/100 cm^2^) of indicator bacteria groups in original drain samples collected from three beef plants (A) and two pork plants (B).

A								
Beef plant sample		Location	APC	TMC	PSY	EB	CF	EC
Plant A	5a	Processing Floor	4.9	5.1	5.0	3.0	2.3	1.1
	6a	Processing Floor	1.2	1.7	1.2	−1.4	0.5	1.1
	7a	Cooler	6.34	6.5	2.6	−1.4	<−1.4	<−1.4
	8a	Cooler	6.1	6.6	6.1	<−1.4	<−1.4	<−1.4
	9a	Cooler	6.7	6.5	5.8	<−1.4	<−1.4	−1.1
	10a	Hotbox	6.2	6.3	2.3	<−1.4	0.9	−0.3
	11a	Hotbox	5.7	6.3	7.0	−1.2	2.7	1.5
	Plant B	1b	Processing Floor	4.8	5.0	3.0	2.3	1.2	0.8
6b		Processing Floor	6.5	7.0	7.9	5.3	0.5	0.1
7b		Cooler	6.23	6.9	7.9	4.9	1.1	<−1.4
8b		Cooler	5.3	7.6	8.6	4.4	0.3	−0.9
9b		Cooler	5.6	6.6	7.9	3.1	1.0	−0.4
10b		Hotbox	3.7	5.4	6.2	3.2	2.0	−0.8
11b		Hotbox	5.7	6.1	6.8	4.1	4.5	<−1.4
Plant C	1c	Processing Floor	7.5	7.7	8.6	6.3	5.1	<−1.4
	2c	Processing Floor	6.8	6.6	7.2	5.5	4.8	<−1.4
	11c	Processing Floor	4.0	3.7	3.9	1.1	−1.4	<−1.4
	12c	Cooler	7.4	7.6	8.6	7.0	<−1.4	<−1.4
	13c	Cooler	7.5	7.7	8.6	6.6	4.1	<−1.4
	14c	Cooler	3.8	7.1	7.4	2.7	<−1.4	<−1.4
	15c	Hotbox	7.7	7.7	8.6	7.2	5.1	<−1.4
	17c	Hotbox	7.7	7.7	8.6	7.1	4.6	<−1.4
	18c	Hotbox	7.7	7.7	8.6	7.6	5.6	<−1.4

B								
Pork plant sample		Location	APC	TMC	PSY	EB	CF	EC
Plant D	1d	Cooler	6.3	7.3	8.0	6.0	5.4	<−1.4
	2d	Cooler	5.8	6.7	7.4	5.1	3.9	<−1.4
	4d	Cooler	5.2	6.7	7.5	4.8	3.1	<−1.4
	5d	Cooler	5.3	6.4	6.9	4.8	4.0	2.6
	6d	Cooler	6.1	6.4	7.4	5.2	6.5	<−1.4
	9d	Fabrication	4.7	5.1	4.7	4.1	2.2	0.6
	12d	Fabrication	5.9	6.3	6.2	5.6	5.3	2.6
	13d	Fabrication	4.7	5.8	5.1	3.9	3.2	1.2
	14d	Fabrication	5.9	5.1	4.6	3.8	2.5	1.2
Plant E	1e	Cooler	5.9	7.4	8.2	4.2	1.2	<−1.4
	2e	Cooler	5.2	7.5	8.0	4.5	<−1.4	<−1.4
	3e	Cooler	5.8	7.6	8.2	4.9	1.1	−0.9
	4e	Cooler	3.4	7.1	7.6	2.0	−0.1	−1.4
	7e	Fabrication	7.3	7.6	6.8	5.7	5.0	4.9
	8e	Fabrication	5.7	6.6	7.2	4.9	3.6	0.6
	9e	Fabrication	4.6	6.5	6.9	4.4	0.1	<−1.4
	11e	Fabrication	5.1	7.2	7.8	4.4	1.6	<−1.4
	12e	Fabrication	3.7	6.1	6.5	2.6	1.6	−1.1

Indicator bacteria measurements include aerobic plate count (APC), total mesophiles count (TMC), psychrophiles (PSY), Enterobacteriaceae (EB), coliforms (CF), and *E. coli* (EC). Values represent log_10_ CFU/100cm^2^ of interior surface or opened floor drain. When no CFU were observed for an organism group the non-detected value was reported as −1.40 log_10_ CFU/100cm^2^, the limit of detection for measurements.

Among the beef plants (Table 2A), samples collected at Plant C had the highest indicator counts overall, and Plant A the lowest. APC, TMC, and PSY were highest in hotbox samples from Plant C (X– = 7.60, 7.60, and 8.60 log_10_CFU/100cm^2^, respectively) and lowest in process floor samples from Plant A (X– = 3.08, 3.43, and 3.10 log_10_CFU/100cm^2^, respectively). While EB and CF were notable in samples collected at Plant C, no EC was detected. In comparison, Plant A had very low concentrations of EB and CF in the samples, but more EC than samples collected at either of the other plants. Among pork plant samples (Table 2B), coolers at Plant E were higher in TMC and PSY than Plant D, but substantially lower in EB, CF, and EC. This difference may have been due to the use of blast chilling at –20°C at Plant D. Blast chilling may have arrested the potential growth of these related groups of bacteria. Levels of organisms recovered from samples collected in the processing areas of the two pork plants were similar except for TMC and PSY being higher at Plant E as well.

Sample 6a collected from the processing floor at Plant A was 3 to 3.5 logs lower in the load of bacteria than the other processing drain sampled at that plant and well lower than at any other plants. Similarly, sample 14c collected from a cooler at Plant C, and sample 10b collected from a hotbox at Plant B had much fewer bacteria present than other similar location drain samples collected at the same and the other plants. Sample 4e collected from a cooler and sample 12e collected from the fabrication area at pork Plant E were lower in bacteria than other pork cooler and fabrication drain samples collected from either plant.

### Mixed biofilm formation and pathogen adherence on contact surface

Bacteria in the enriched and pooled drain samples attached on both contact surfaces and the total enumerable bacteria incubated at 7°C ranged from 5.1 to 6.5 and 5.1 to 7.1 log_10_ CFU/chip on SS and tile surfaces, respectively. The two pooled pork plant samples from fabrication rooms overall developed higher biofilm mass at 15°C, reaching 7.0 to 7.2 log_10_ CFU/chip on the two types of surfaces. However, no significant difference was detected in the total bacterial cell density among the pooled drain samples and between the two surface types.

**Figure 1:**
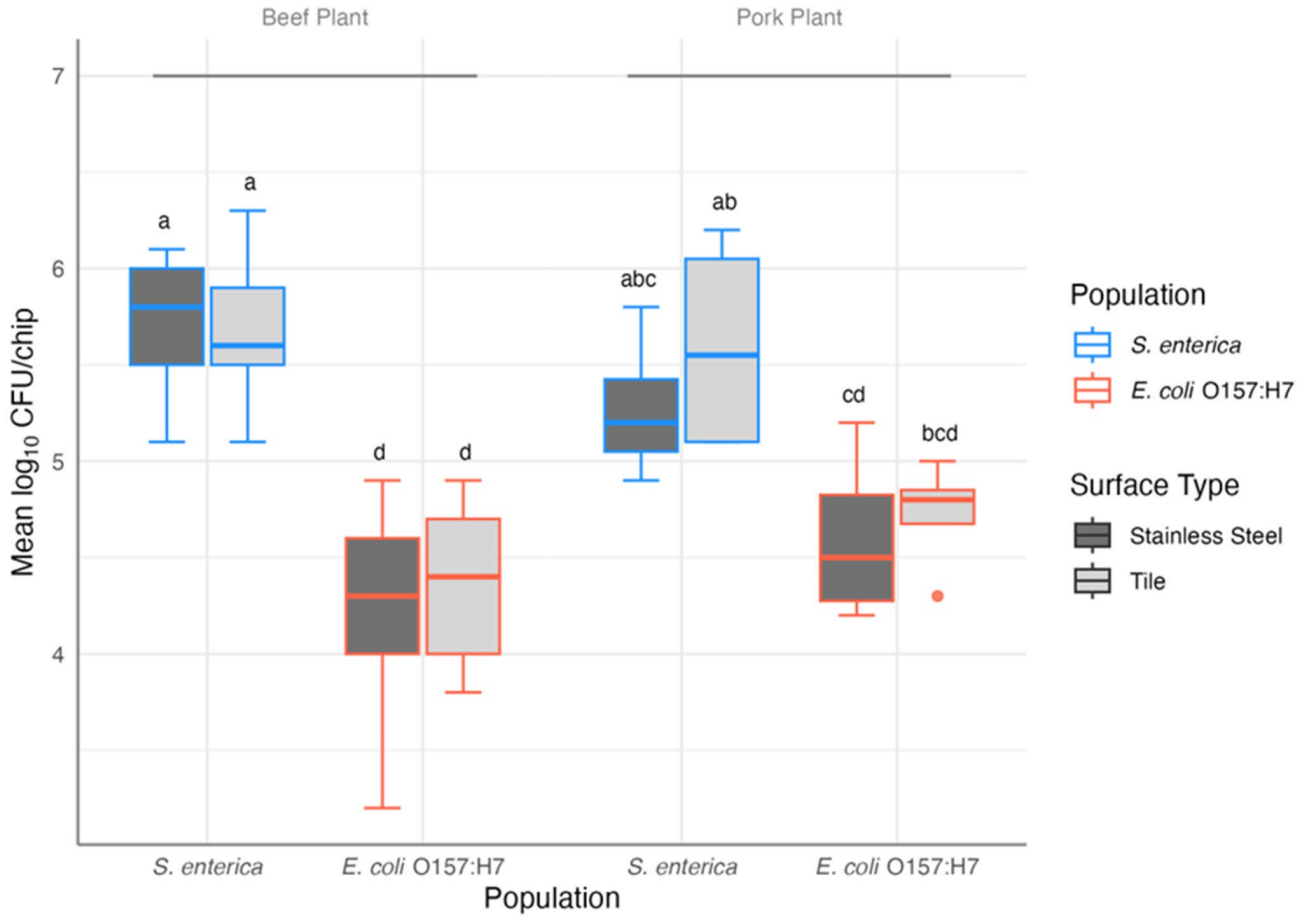
Results of the three-way ANOVA and post-hoc comparison of the mean log_10_ CFU/chip of *S. enterica* and *E. coli* O157: H7 in mixed biofilm samples collected from beef (*n* = 9) and pork (*n* = 4) plants on stainless steel and tile surfaces. Letters provide significant contrasts.

Notably, significant adherence of *E. coli* O157: H7 and *S. enterica* in the multispecies mixture on both contact surfaces was observed. Cell densities of the attached *S. enterica* and *E. coli* O157: H7 ranged from 4.9 to 6.3 and 3.2 to 5.2 log_10_ CFU/chip, respectively. The three-way ANOVA revealed significant difference between the attached *S. enterica* and *E. coli* O157: H7 cell densities (*F*_(1,44)_ = 104.240, *p* = 3.51×10^−13^), with *S. enterica* being more abundant. Contact surface materials and meat plant types did not differ significantly in pathogen adherence of either species (F_(1,44)_ = 0.59 and 0.26, *p* = 0.45 and 0.61, respectively). However, the interaction of species and plant type was a significant predictor of pathogen persistence (F_(1,44)_ = 5.471, *p* = 0.024): in the pork plant, the presence of *S. enterica* and *E. coli* O157: H7 in the multispecies mixtures on any surface type did not differ significantly (all post-hoc *p* > 0.05) except for *S. enterica* on tile vs. *E. coli* O157: H7 on SS (post-hoc *p* = 0.029; Figure 1).

### Pathogen survival and recovery after sanitization

The multi-component sanitizer exhibited high effectiveness against pathogen cells harbored in the multispecies communities when applied with the foam coverage method, reducing viable cells of both pathogens in each sample to a level lower than the limit of detection (2.2 log_10_ CFU/chip). The treatment further inhibited post-sanitization recovery growth and prevalence of both pathogens in all beef plant samples on SS and tile surfaces. However, positive prevalence of *E. coli* O157: H7 was observed in all four pork plant samples on the SS surface. On the tile surface, *S. enterica* and *E. coli* O157: H7 positive prevalence was detected in one (E2) and two (D1, E2) pork plant samples, respectively, after overnight enrichment of the treated samples (Table 1).

The multi-component sanitizer fog treatment was also effective against *E. coli* O157: H7 similar to the foam treatment. *E. coli* O157: H7 cells were reduced to the non-detectable level in all samples and no post-sanitization prevalence was detected. However, *S. enterica* was more tolerant to the fog treatment. Viable *S. enterica* cells were detected in all four pork plant samples and three beef plant samples (A2, B3, and C3). No *S. enterica* recovery was detected in the other six beef plant samples that had no enumerable *S. enterica* cells immediately after the fog treatment (Table 1).

### SEM analysis

**Figure 2:**
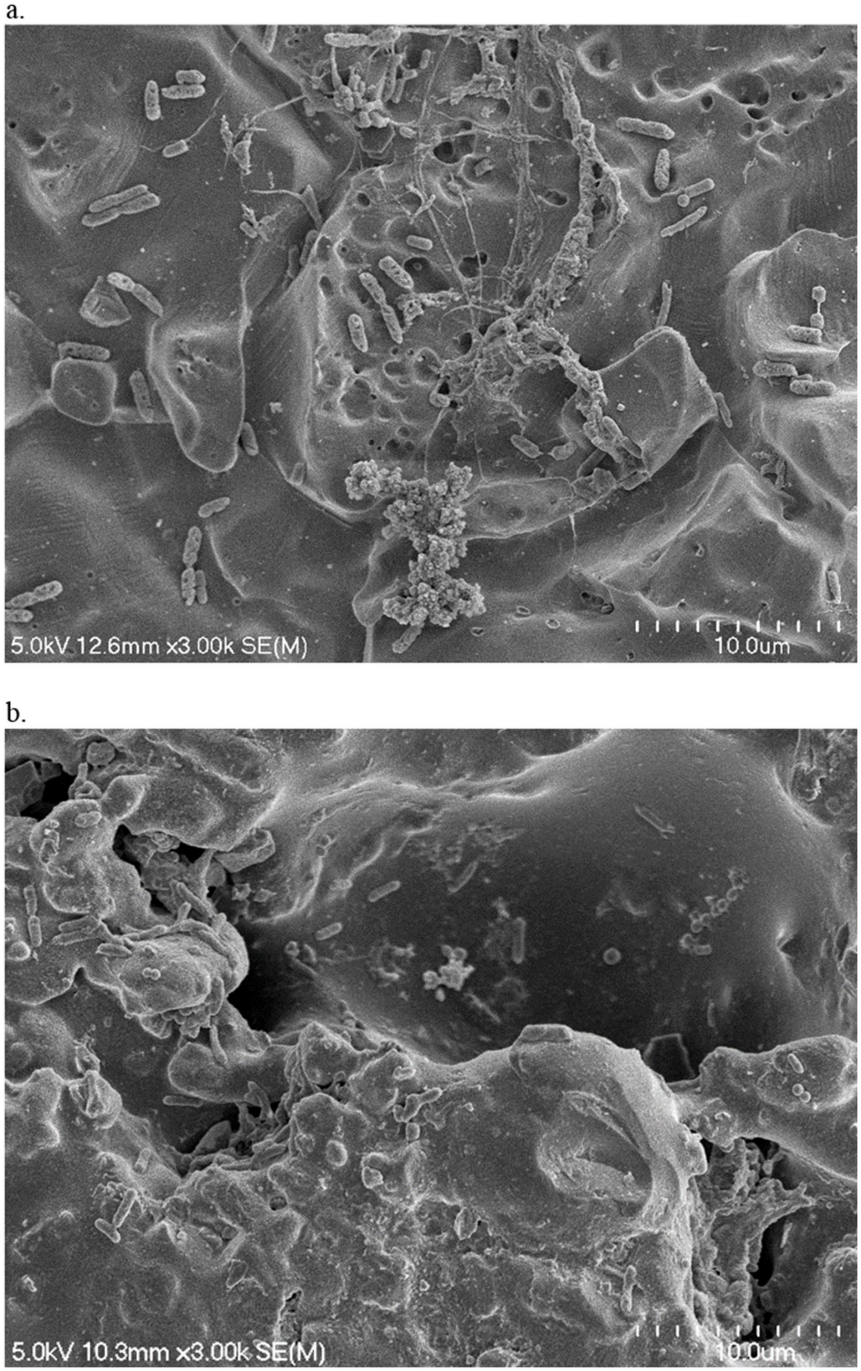
Scanning electron micrographs of sterile water-treated SS **(a)** and tile **(b)** chips that were incubated with cooler drain sample A2 for 5 days at 7°C. Magnification 3.0 k.

On both contact surfaces incubated with the pooled floor drain samples at 7°C or 15°C, individually colonized bacterial cells or aggregated microcolonies well connected to the surfaces by the production of the EPS structures were visualized (Figure 2). Even though similar amounts of total attached bacteria were measured on both contact surfaces, the surface material types affected the morphology of the attached bacteria and the microcolonies. On the relatively smooth SS surface as shown in the SEM image (Figure 2a), different cell types and microcolony morphologies were observed as expected since multiple bacterial species were present in the samples that can exhibit distinct structural features and matrix compositions. As a result, the attached bacteria exhibited a diverse morphology such as dense clusters of bacteria in cauliflower/broccoli shapes with well-expressed cell surface structures, or long filamentous appendages extending from the aggregate surface that connected the different types of bacteria to each other and to the SS surface. In comparison, the tile chips exhibited higher surface roughness with small holes, dents, and trenches (Figure 2b). The irregularly rough surface texture provides the reservoir for cell adhesion and attachment as more individually scattered/adhered cells or small bacterial clusters with less EPS expression were attached to or embedded underneath the dents and trenches.

### Metagenomic analysis of the natural microbial communities at the plants

The 16S rRNA sequencing analysis of the 13 pooled floor drain samples yielded a total of 2,560,220 raw reads that were processed using Qiime2. The maximum reads were recorded in the beef plant cooler sample A2 with 242,905 reads and the minimum in the beef plant processing floor sample C1 with 120,678 reads. An average of 196,940 reads were present in all samples. Pre-processing with DADA2 yielded a total of 2,210,214 (86.32%) cleaned reads.

**Figure 3:**
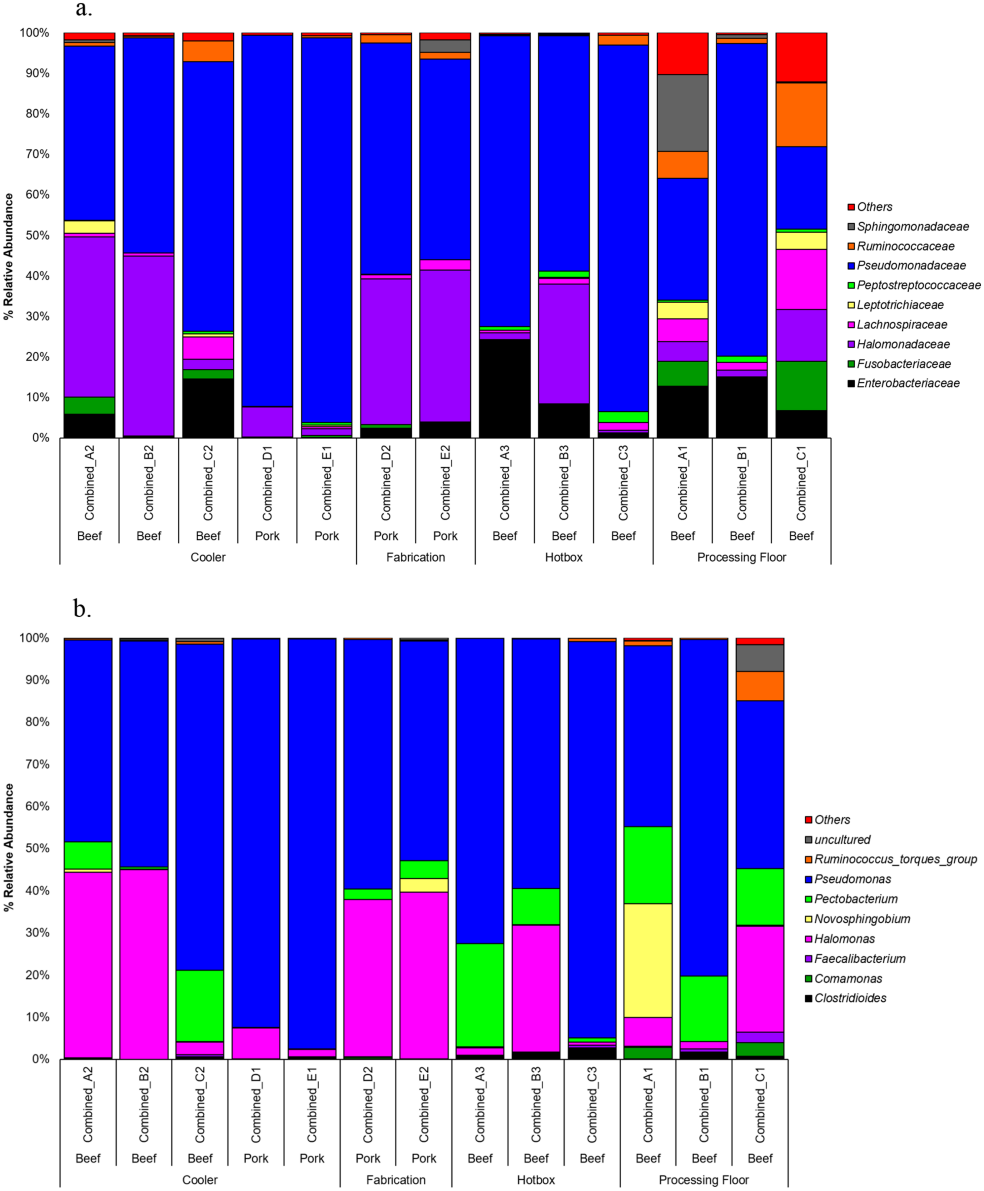
Percent relative abundance at family level **(a)** or genera level **(b)**.

Analysis of percent relative abundance at bacterial family and genera levels showed that *Pseudomonadaceae* was the most abundant family ranging from 15 to 95%. The pooled cooler drain sample from pork plant E had the highest abundance of *Pseudomonadaceae*, while the processing floor sample (C1) of beef plant C had the lowest (Figure 3). Successively, the genus *Pseudomonas* of the *Pseudomonadaceae* family was highly dominant, with the same percentage of abundance and samples as *Pseudomonadaceae*. Furthermore, *Pseudomonas* was abundant in the pooled hotbox sample A3 from beef plant A at 67%. *Halomonadaceae* was the second most abundant family, with a range of 1 to 35%, and the greatest abundance in the cooler sample B2 of the beef plant B. Pork plant fabrication sample D2 observed 28% of *Halomonadaceae*. Genus *Halomonas* had the same abundance as *Halomonadaceae* in the same samples mentioned above. The third family, *Enterobacteriaceae*, was mildly dominant across all samples, with the highest abundance of 23% recorded in the hotbox sample A3 of beef plant A. There was no evidence of *Enterobacteriaceae* in the cooler samples from beef plant B (sample B2) or pork plants D and E (samples D1 and E1).

**Figure 4:**
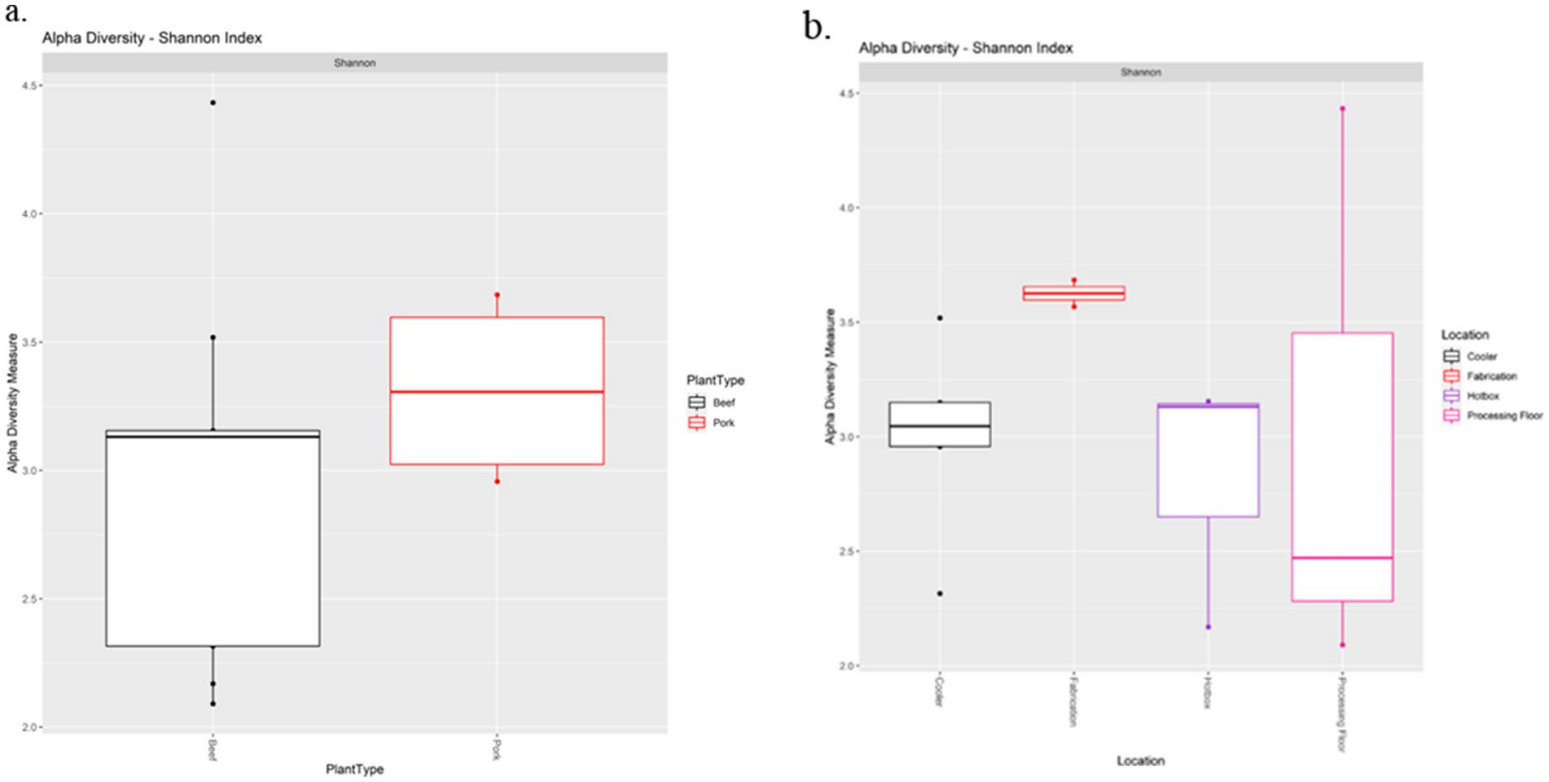
Alpha diversity analysis of processing plants **(a)** and drain locations **(b)** based on the Shannon diversity index metric.

**Figure 5:**
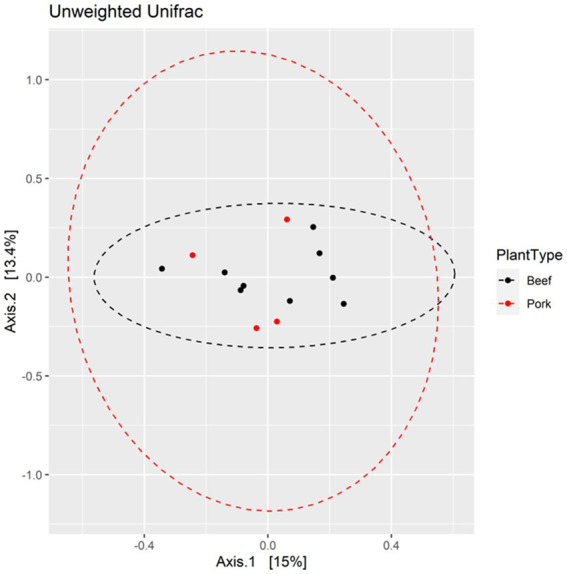
PCoA plots displaying beta diversity between pork and beef plants based on Unweighted Unifrac metric.

The Shannon diversity index metric was used to perform alpha diversity analysis between the beef and pork samples, as well as among the locations. There was no significant difference in Shannon index values between the beef and pork plants (Kruskal-Wallis test, *χ*^2^ = 0.85714, *p* = 0.3545; Figure 4a). Similarly, all locations had a Shannon index greater than 2.0, with no significant difference in diversity based on the Wilcoxon rank sum test (Figure 4b). The Unweighted Unifrac metric was used to analyze beta diversity. PCoA plots show no distinct clustering of samples (Figure 5) from the beef and pork plants, which is supported by PERMANOVA that yielded no statistically significant results (*F* = 0.9582, *p* = 0.548).

## Discussion

In meat processing plants, multispecies mixed biofilms can be formed in difficult-to-clean areas such as ceilings, sinks, pipes, hard-to-reach corners of equipment, contact surfaces, and the back of conveyor belts. These areas attract and support biofilm development and persistence due to poor accessibility during sanitization (Mathijssen et al., 2016). We observed various levels of the general indicator microorganisms in the original drain samples collected from distinct areas at the beef or pork plants, likely as the result of the different levels/types of the processing activity, operation/cleaning routines, and the subsequent liquid runoff near the areas.

The enriched and pooled drain samples were all able to attach effectively onto the common contact surfaces at a similar level. However, cell density of the adhering total bacteria as well as the inoculated pathogen cells were relatively low, especially for the samples incubated at 7°C. Mature and dense biofilm structures were not detected with SEM. Rather, the observed bacterial populations mainly consisted of individually attached cells, cellular aggregates and nascent microcolonies with cell surface appendages implicated in initial adhesion. This is likely due to the low temperature that inhibited active replication of most bacterial species in the samples. Also, since the natural floor drain samples were applied in the present study, the actual inoculated cell densities of the various species, the growth competition within the multispecies mixtures, and the relatively short incubation time period may all affect mixed biofilm development and maturation. Nevertheless, it is important to notice in our study that even at the low temperature (7°C) often seen in the processing plants, significant amount of both pathogens was present in the attached multispecies mixture, indicating that the low temperatures commonly maintained at meat plants are not able to effectively prevent pathogen persistence within the multispecies microbial community so there could be persistent pathogen cells on contact surfaces that can contaminate the products.

To address such challenges in the processing areas and on contact surfaces, besides the common sanitizer liquid spray and immersion treatment, multi-component sanitizers have been more commonly applied as foam or fog to cover the large-scale processing facilities and gain access to all areas difficult to reach for thorough and extended exposure. In the present study, we applied the multi-component sanitizer with either the foam or fog method following the manufacturer’s instruction to simulate the procedure commonly used in the industry.

Our previous studies (Wang et al., 2020, 2021) showed that the novel multi-component sanitizer products containing the combined chemical reagents could function effectively against single-species biofilms formed by *S. enterica* or *E. coli* O157: H7. The present study further demonstrated that this multifaceted approach was also effective in inactivating the pathogens presented within the environmental microbial community which may potentially provide protection for the pathogens once they integrate into the multispecies mixture. Our previous studies also showed that single-species biofilms formed by *S. enterica* strains were overall more tolerant than those by the *E. coli* O157: H7 strains when treated with the multi-component sanitizers (Wang et al., 2020, 2021). In the present study, we obtained similar observations with fog treatment targeting the two types of pathogens in the mixed community. However, even though the more effective foam treatment reduced both pathogens to the non-enumerable level, the *E. coli* O157: H7 cells exhibited more recovery growth in pork plant samples. The longer exposure time of the fog treatment (3 h) and the sensitivity of the different pathogen cells as well as the companion bacterial cells toward the surface drought following the treatment might influence pathogen susceptibility and recovery, which requires further investigation. Nevertheless, such observation suggests that consecutive treatments combining the different application methods (foam coverage followed by fog treatment) can be an effective means to improve the results, especially when multiple types of pathogens may simultaneously be present in the multispecies bacterial community at the meat plants.

Contact surface topography may affect biofilm morphology. Previous studies tested various single-species bacteria and observed biofilm morphology differences on either rough or smooth contact surfaces. For instance, Wu et al. (2018) reported that cells of *Pseudomonas aeruginosa* and *Staphylococcus aureus* tended to colonize individually as single cells on rough SS surface but as clusters of aggregated cells on electropolished smooth SS surface. Similarly, it was reported that on rough titanium surface *S. aureus* cells were scattered and formed small clumps (2–4 bacteria) but on polished titanium and SS surfaces the bacteria colonized in large aggregates (Harris et al., 2007). In addition, contact surface topography may also influence the expression of bacterial cell adhesins (Rizzello et al., 2012, 2013). Interestingly, in the present study using multispecies mixed bacterial cultures, we obtained similar observations in terms of microcolony morphology and bacterial EPS expression between smooth SS surfaces and rough tile surfaces. This is likely due to the fact that the rough topography provides scaffolds easier for the single cells to attach and embed inside the holes/dents (Figure 2b), but larger cell aggregates with strong EPS connections are required for the bacteria to attach on smooth surface (Figure 2a). The various genres of bacteria may behave differently in response to contact surface texture and other environmental conditions, as Zheng et al. reported (Zheng et al., 2021) that contact surface topography might affect bacterial colonization selectively depending upon the species. Therefore, the presence and percentage of the various bacterial species in our drain samples and their unique responses to the different surface topography warrants further investigation.

Sanitizer efficiency may also be related to the difference in contact surface texture that may affect chemical exposure and penetration. The effect of contact surface topography on bacterial colonization and stress tolerance has been investigated previously. For instance, it was reported that a surface with a rough texture may facilitate biofilm development by providing a larger surface area for bacterial attachment, reducing the shear forces, protecting bacteria from detachment, and trapping residual nutritional particles for cell survival and proliferation (Yoda et al., 2014; Bollen et al., 1996). In the present study, the bacteria attached on the tile surface overall exhibited a higher tendency for survival and recovery growth after the multi-component sanitizer treatment compared to those on SS surface (data not shown), which might be related to the different surface topography. As shown in the SEM images (Figure 2), compared to the SS chips the tile surface contains a rougher topography. The irregular rough surface may not only function as a scaffold for bacterial attachment but also provide a shelter covering the bacterial cells underneath the holes, dents, cavities, and trenches. Thus, it is more difficult for the sanitizer foam or fog to reach and penetrate the hidden cell aggregates to achieve complete inactivation and removal of the surface adhered microbial community.

The composition of natural mixed biofilms at different processing facilities may vary depending upon the plant processing activities, animal types and sources, local microbial community, and the selective pressure caused by the daily cleaning practice and sanitization reagents (Lorenzo et al., 2018; Galié et al., 2018). In the present study, metagenomic analysis of the microbial composition of the pre-sanitization microbial communities isolated from the processing facilities revealed no significant difference in alpha and beta diversity between the beef and pork plants. Our recent shotgun sequencing study (Palanisamy et al., 2023) analyzing drain samples isolated from these three beef plants also showed that even though the abundance of bacteria varied, similar microbiomes existed in the three plants regardless of how far apart they are located in the geographic distance and the different sampling time points (2027–2018 & 2021), suggesting that geography is not a good predictor for the microbial community compositions at different food processing facilities.

It should also be noted that the sample size of the present study for the comparison of microbial communities between the beef and pork processing facilities is relatively limited (3 beef plants vs. 2 pork plants), therefore further investigation is needed to include large sample sizes from additional plants of each type. More samples should also be collected at various time points as the composition and relative abundances of the bacteria may change over time due to various environmental conditions. Nevertheless, our data suggests that attempts to use metagenomic data as a means to track beef and pork items to points of processing or origin (Doyle et al., 2017) lack support.

Interestingly, the adhered pathogen cells in the pork plant samples overall exhibited higher tolerance to sanitization in terms of survival and recovery compared to those in the beef plant samples, as shown by the enumerable *S. enterica* cells after fog treatment and the *E. coli* O157: H7 positive prevalence after foam treatment (Table 1). Despite the similar community compositions between the beef and pork plant samples, species percentages and the patterns of relative abundance of bacterial families varied among the samples. It has been known that the various natural bacterial species and their ability to form mixed biofilms can affect the stress tolerance of the pathogens integrated within the community. For instance, the presence of *Pseudomonas* in dual-species biofilms was found to enhance stress tolerance and survival of the companion *Salmonella* cells (Pang et al., 2017, 2020; Pang and Yuk, 2018). Interestingly, *Pseudomonadaceae* was the most abundant family in the examined samples and the potential correlation between its high presence and *Salmonella* tolerance in certain pork plant samples requires further investigation.

Since the multi-component sanitizer in this study is highly effective and, in most cases, completely inactivated the multispecies samples containing the pathogens with low to no bacterial recovery growth after enrichment, we are unable to genetically analyze these samples for the post-sanitization bacterial community shift. However, to understand the synergistic and/or antagonistic interactions within the multispecies community that may either promote or inhibit pathogen survival, 16S rRNA sequencing of the drain samples with the co-inoculated pathogen strains and data correlation network analysis might provide valuable insight into the positive or negative correlations among the microbial community members within the ecosystem. Such analysis and modeling can identify associations among the abundance of taxa in the microbial communities harboring the pathogen and also identify potential environmental keystone species or combinations of species that can impact pathogen survival.

## Conclusion

Meat processing plants harbor a wide variety of multispecies microorganisms, often persisting as mixed biofilms, that may affect sanitizer effectiveness against the targeted pathogens once they are integrated into the mixed communities. The present study demonstrated that the common foodborne pathogens can efficiently persist within the multispecies microbial community even under preventive low temperatures, posing a serious risk to meat safety. A multi-component sanitizer with various application methods was effective against pathogens harbored in the mixture and prevented post-treatment prevalence. However, the interactions within the multispecies microbial community and their impact on pathogen tolerance and persistence require further investigation. In addition, other factors such as temperature and contact surface texture on pathogen adherence and survival should be taken into consideration as well while evaluating treatment effectiveness. Therefore, sanitization protocols designed for the different types of processing facilities should be analyzed on a case-by-case basis.

## Data Availability

Raw data (fastq) are not publicly available, as they originate from commercial meat processing plants, and the authors are obliged to maintain confidentiality, preventing the public deposition of these sequences. However, the authors are prepared to share the data upon reasonable request through secure file sharing. Data access is subject to a non-disclosure agreement (NDA) with the meat processors, and those interested must agree to the same terms. Requests for dataset access should be directed to the corresponding author.

## Footnotes

^1^ https://www.bioinformatics.babraham.ac.uk/projects/fastqc/

^2^ https://qiime2.org/

^3^ https://data.qiime2.org/2021.11/common/silva-138-99-nb-classifier.qza
