# Supplementary material for: Characterization of multispecies microbial communities at beef and pork processing plants and their impact on pathogen stress tolerance
Source: Front Microbiol. 2025 Jul 2;16:1605719. doi: 10.3389/fmicb.2025.1605719 (PMC12263626; doi:10.3389/fmicb.2025.1605719)

Supplement Figure 1. Sequence statistics of raw and filtered reads processed using Qiime2 and DADA2.

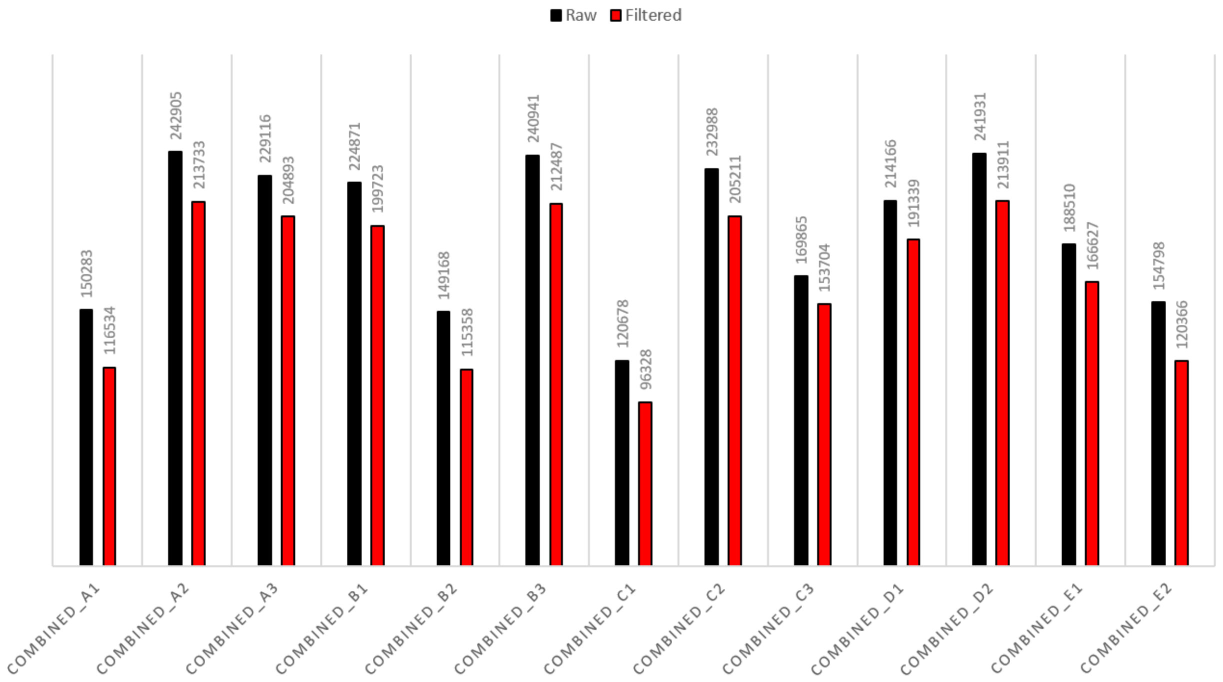

Supplement: Supplementary file 1 [file Data_Sheet_1.PDF]
