# Supplementary material for: Characterization of multispecies microbial communities at beef and pork processing plants and their impact on pathogen stress tolerance
Source: Front Microbiol. 2025 Jul 2;16:1605719. doi: 10.3389/fmicb.2025.1605719 (PMC12263626; doi:10.3389/fmicb.2025.1605719)
Supplement: Supplementary file 2 [file Data_Sheet_2.PDF]

Pool	Plant	Plant Type	Location	Month	sample	Floor description	Drain description	Fig
A1	A	Beef	Fabrication	June	5a	bare rough concrete	Side trap with basket	A
					6a	bare rough concrete	Side trap w/ basket Stainless Steel	B
A2	A	Beef	Cooler	June	7a	non-slip concrete	Side trap with basket	A
					8a	non-slip concrete	Square industrial w/o basket; adjacent cleanout	C
					9a	bare rough concrete	Round Industrial w/o basket	D
A3	A	Beef	Hotbox	June	10a	bare rough concrete	Side trap with basket	A
					11a	bare rough concrete	Square industrial w/o basket	E
B1	B	Beef	Fabrication	July	1b	smooth concrete	Trench	F
					6b	smooth concrete	Square industrial with basket	H
B2	B	Beef	Cooler	July	7b	bare rough concrete	Trench w/ adjacent clean out	G
					8b	bare rough concrete	Trench w/ adjacent clean out	G
					9b	bare rough concrete	Trench w/ adjacent clean out	G
B3	B	Beef	Hotbox	July	10b	bare rough concrete	Trench	F
					11b	bare rough concrete	Trench	F
C1	C	Beef	Fabrication	August	1c	concrete and orange brick tile	Side trap w/ basket Stainless Steel	I
					2c	concrete and orange brick tile	Side trap w/ basket Stainless Steel	I
					11c	concrete and orange brick tile	Side trap w/ basket Stainless Steel	I
C2	C	Beef	Cooler	August	12c	bare rough concrete	Round Industrial w/o basket	D
					13c	bare rough concrete	Round Industrial w/o basket	D
					14c	bare rough concrete	Round Industrial w/o basket	D
C3	C	Beef	Hotbox	August	15c	bare rough concrete	Square industrial w/o basket	E
					17c	bare rough concrete	Round Industrial w/o basket	D
					18c	bare rough concrete	Round Industrial w/o basket	D
D1	D	Pork	Cooler	June	1d	orange non-slip concrete	Square industrial w/o basket	E
					2d	orange non-slip concrete	Square industrial w/o basket	E
					4d	orange non-slip concrete	Square industrial w/o basket	E
					5d	orange non-slip concrete	Side trap w/o basket	J
					6d	orange non-slip concrete	Side trap w/o basket	J
D2	D	Pork	Fabrication	June	9d	bare rough concrete	Square industrial with basket	L
					12d	orange non-slip concrete	Square industrial w/o basket	K
					13d	orange non-slip concrete	Square industrial w/o basket	K
					14d	orange non-slip concrete	Square industrial w/o basket stainless grate	M
E1	E	Pork	Cooler	July	1e	non-slip concrete	Round Industrial w/o basket Stainless steel	N
					2e	non-slip concrete	Round Industrial with basket Stainless steel	O
					3e	non-slip concrete	Round Industrial with basket Stainless steel	O
					4e	non-slip concrete	Round Industrial with basket Stainless steel	O
E2	E	Pork	Fabrication	July	7e	non-slip concrete	Round Industrial w/o basket	N
					8e	non-slip concrete	Side trap w/o basket	J
					9e	non-slip concrete	Square industrial w/o basket	P
					11e	non-slip concrete	Round Industrial w/o basket Stainless steel	N
					12e	non-slip concrete	Side trap w/ basket Stainless Steel	Q
