# Supplementary material for: Characterization of multispecies microbial communities at beef and pork processing plants and their impact on pathogen stress tolerance
Source: Front Microbiol. 2025 Jul 2;16:1605719. doi: 10.3389/fmicb.2025.1605719 (PMC12263626; doi:10.3389/fmicb.2025.1605719)
Supplement: Supplementary file 4 [file Data_Sheet_4.PDF]

Supplement Table 2. The presence of *S. enterica* and *E. coli* O157:H7 cells ($\log_{10}$ CFU/chip) in the multispecies microbial communities attached on stainless steel or tile surface.

Sample Name	SS		Tile	
	S. enterica	*E. coli* O157:H7	*S. enterica*	*E. coli* O157:H7
A1	6.0 (0.2)	4.3 (0.4)	5.9 (0.1)	4.7 (0.2)
A2	5.2 (0.2)	4.0 (0.4)	5.1 (0.3)	4.0 (0.2)
A3	5.8 (0.8)	4.4 (0.0)	5.5 (0.1)	4.4 (0.1)
B1	5.8 (0.4)	4.7 (0.1)	6.0 (0.2)	4.7 (0.2)
B2	5.1 (0.4)	4.0 (0.3)	5.3 (0.2)	3.9 (0.2)
B3	5.5 (0.1)	4.9 (0.4)	5.5 (0.4)	4.3 (0.3)
C1	6.0 (0.2)	4.2 (0.1)	5.6 (0.1)	4.5 (0.1)
C2	6.1 (0.1)	4.6 (0.3)	6.3 (0.2)	4.9 (0.1)
C3	5.7 (0.1)	3.2 (0.3)	5.6 (0.1)	3.8 (0.3)
D1	5.3 (0.1)	4.3 (0.2)	5.1 (0.4)	4.8 (0.6)
D2	4.9 (0.5)	5.2 (0.0)	6.0 (0.1)	5.0 (0.2)
E1	5.1 (0.3)	4.2 (0.1)	5.1 (0.4)	4.8 (0.0)
E2	5.8 (0.3)	4.7 (0.7)	6.2 (0.2)	4.3 (0.1)
